# Relationship between the Ingestion of a Polyphenol-Rich Drink, Hepcidin Hormone, and Long-Term Training

**DOI:** 10.3390/molecules21101333

**Published:** 2016-10-08

**Authors:** Débora Villaño, Cristina Vilaplana, Sonia Medina, Francisco Algaba-Chueca, Roberto Cejuela-Anta, Jose Miguel Martínez-Sanz, Federico Ferreres, Angel Gil-Izquierdo

**Affiliations:** 1 Department of Food Science and Technology, CEBAS-CSIC, Campus de Espinardo 25, Espinardo, Murcia 30100, Spain; dvillano@ucam.edu (D.V.); christinavilaplana@gmail.com (C.V.); falgabachueca@gmail.com (F.A.-C.); federico@cebas.csic.es (F.F.); 2 Faculty of Education, University of Alicante, Campus de Sant Vicent del Raspeig, Alicante 03690, Spain; roberto.cejuela@ua.es (R.C.-A.); josemiguel.ms@ua.es (J.M.M.-S.)

**Keywords:** polyphenols, hepcidin, iron, exercise, juice

## Abstract

The effects of polyphenol-rich foods on the iron status of athletes, as well as the effect of physical training on the hormone hepcidin, implicated in iron metabolism, are not clear. We investigated the influence on iron metabolism of a long-term training intervention of 120 days, measuring the hepcidin concentration in the plasma of 16 elite triathletes, and the effect of the ingestion of 200 mL of either aronia-citrus juice or a placebo drink for 45 days, in a crossover design. The highest plasma hepcidin concentrations were observed at the beginning of the study (116 ± 63 nM) and levels steadily decreased until the end of the intervention (final value 10 ± 7.5 nM). Long-term training might reduce inflammation and, hence, could be responsible for the decrease in hepcidin in triathletes. Polyphenols from aronia-citrus juice did not interfere in iron absorption, as we did not observe significant differences between the intake of the placebo drink or juice with regard to hepcidin levels. Further studies are required to ascertain the time and conditions necessary to restore hepcidin levels, which reflect the iron status of triathletes.

## 1. Introduction

Hepcidin is a peptide hormone synthesized mainly in the liver and the active form consists of 25 amino acids. Some studies have demonstrated the relationship between hepcidin and iron. Nicolas et al. observed a severe iron overload in mice that were knockout for the gene encoding hepcidin [1]. It has also been shown that hepcidin is regulated by hemojuvelin, a protein produced in the liver and whose gene expression is responsible for juvenile hemochromatosis, causing excessive accumulation of iron in different organs, mainly in the liver [2], and promoting the development of certain infectious diseases [3]. The maintenance of iron levels involves regulation of iron absorption from the diet and its storage in hepatic and splenic macrophages. During absorption, iron is taken up by enterocytes, where it will bind to ferroportin or be incorporated into intracellular ferritin for storage [4,5]. Hepcidin has been shown to have its main role in iron homeostasis, as it regulates transmembrane iron transport. It binds to the cell surface receptor, ferroportin, which is then internalized and digested by lysosomes. Ferroportin enables iron flux from enterocytes, hepatocytes, and macrophages into blood. Hence, the decrease in ferroportin levels in enterocytes due to hepcidin action leads to decreased iron absorption in hepatocytes and macrophages, due to a reduction in iron recycling from senescent erythrocytes, and to low serum iron levels.

Some studies have shown the relationship between physical activity and iron absorption. In rats it has been observed that intense exercise decreases the absorption of iron [6]. Studies in humans have shown increased prevalence of a deficient iron status in athletes that may affect up to one-third of the population of athletes [7]. Inadequate body iron stores may decrease physical performance. This effect should not be confused with sport anemia—which is more frequent in endurance athletes, being named “runner′s anemia” or “swimmer′s anemia”. In this case, training produces a rapid expansion of plasma volume and a slow expansion of the red blood cells, which explains the transient decrease in the hematocrit, especially in the early stages of training [8].

The deficient iron status mentioned before has been attributed to several factors, including increased destruction of red blood cells via foot-strike, hemolysis, inadequate dietary intake of iron, iron loss via sweating, and gastrointestinal blood loss [9]. In recent years, the discovery of the iron regulatory hormone hepcidin revealed another possible cause of iron deficiency. Furthermore, it has been observed that various factors, such as hypoxia, inflammation, and iron level, are able to regulate hepcidin levels [10]. A relationship between hepcidin and inflammation has been described, with a response similar to the acute phase of inflammation frequently observed after acute exercise. In response to inflammation, the hepcidin concentration increases in order to reduce or limit the iron available to invading pathogenic microorganisms [11]. Some studies have investigated the connections between inflammatory processes and hepcidin levels [12,13]. In 2006, Wrighting and Andrews demonstrated that the inflammatory cytokine interleukin-6 (IL-6) regulated hepcidin through induction and subsequent promoter binding of the signal transducer and activator of transcription 3 (STAT3). In fact, IL-6 is the main stimulator of hepcidin synthesis [3,12,13]. In athletes, the use of dietetic supplementation, primarily to prevent deleterious effects of exercise, is very common [14].

Phytochemical compounds, such as flavonoids, might provide health benefits with regard to sport, but studies on the efficiency of polyphenol-rich foods in relation to iron absorption in this type of population are scarce. We have previously observed an increase in the bioavailability of flavanones from a citrus-based juice after exercise, which could provide positive effects for health [15]. On the other hand, limited information is available on the impact of nutritional intervention on the novel hormone hepcidin and the ferric status of athletes.

The aim of the present study is to investigate a citrus-based juice, rich in polyphenols and vitamin C, along with long-term exercise affect iron status, considering changes in serum hepcidin levels, in a sample of elite triathletes.

## 2. Results

**Figure 1:**
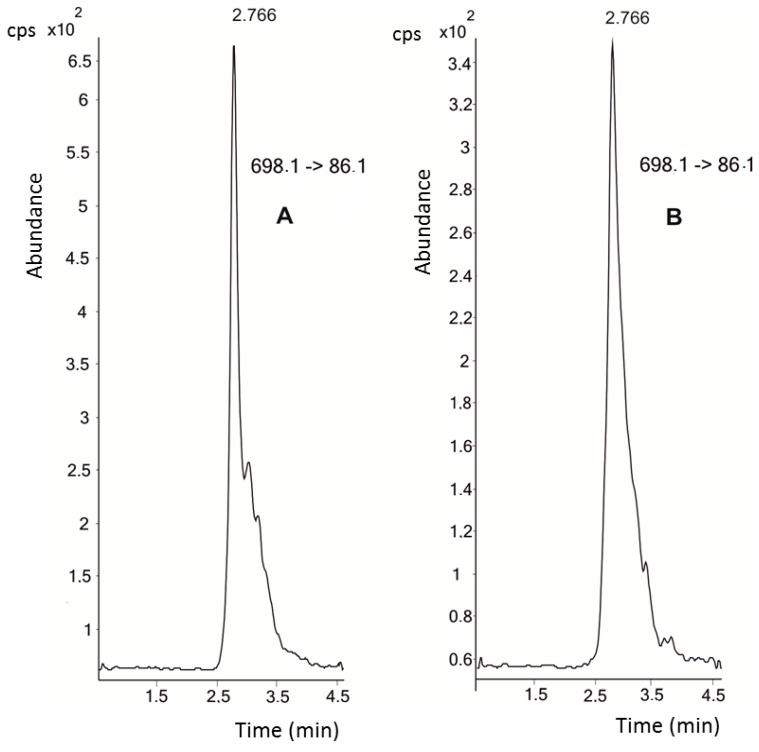
(**A**) Hepcidin standard at a concentration of 10 nM; and (**B**) endogenous hepcidin in a plasma sample.

The mass spectrometer (MS) parameters (ion optics; capillary exit voltage) were optimized at a hepcidin concentration of 10 μM. The fragmentor and collision energy conditions were calculated for [M + 4H]^4+^ *m/z* 698.1, *m/z* 703.2, and [M + 5H]^5+^ *m/z* 558.7, *m/z* 562.8 for hepcidin and the [^13^C_18_, ^15^N_3_]-hepcidin standard, respectively. These were found to be the most intense ions, in accordance with Bansal et al. [16]. We selected the transitions 698.1 → 86.1 and 562.8 → 70.2 to quantify hepcidin levels in the plasma samples of triathletes. Figure 1 shows the chromatograms of both the 10 nM hepcidin standard and the endogenous hepcidin present in the samples. The highest plasma concentrations of hepcidin were recorded at the beginning of the study, with a mean value of 116.6 ± 63.3 nM (baseline control with mild exercise and a mean ECO (*Equivalentes de Carga Objetiva*, Objective-Load Scale) value of 37.5). Afterwards, hepcidin levels decreased steadily until the end of the study period. In fact, after two weeks of intensified training (control 2, mean ECO value of 1008) a mean hepcidin concentration of 78.7 ± 71.1 nM was found (Table 1). The levels markedly decreased during the nutritional intervention with drinks, irrespective of the treatment (mean values of 15.2 nM for the placebo drink and 29.4 nM for the aronia-citrus juice drink). The hepcidin concentrations diminished to a mean value of 10 nM in the final control samples.

The confidence intervals show a high variability in the results due to inter-individual differences. The Levene test did not show homogeneity of variance and, hence, we performed a non-parametric statistical analysis.

**Figure 2:**
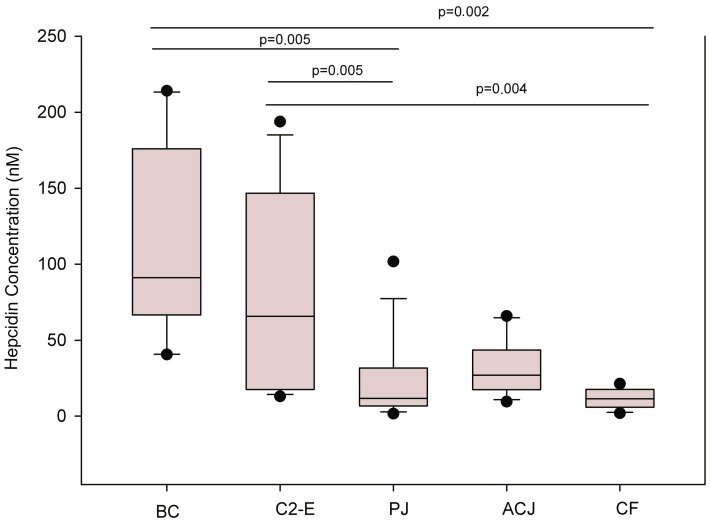
Hepcidin levels (nM) determined in plasma samples of triathletes following the training and nutritional intervention program. Box plots with quartiles (upper values, 75%; median, 50%; and lower values, 25%). Friedman′s ANOVA and post hoc analysis with Wilcoxon signed-rank tests (with a Bonferroni correction) were conducted (*p* < 0.005).

A description of the distribution of data is shown in the box plots diagram (Figure 2).

The Wilcoxon test revealed that the changes in hepcidin concentration were of statistical significance for the placebo treatment and baseline control (*p* = 0.005) and for the control 2-exercise (*p* = 0.005). The final control group showed statistical significance for the baseline control (*p* = 0.002) and for the control 2-exercise (*p* = 0.004). The aronia-citrus juice (ACJ) treatment produced a slight difference when compared to the baseline control and control 2, although non-significant differences were observed between the two test drinks.

## 3. Discussion

We have determined the effect of exercise and the consumption of a polyphenol-rich beverage on hepcidin levels in triathletes. Our data show a decrease in plasma hepcidin concentrations in the triathletes after a training schedule. Major regulation of hepcidin levels results from the high production of pro-inflammatory cytokines, such as IL-6, hypoxic conditions, and plasma iron levels [10]. Concerning exercise, the intensity of training is a crucial factor influencing iron metabolism and hepcidin. Some authors have investigated the influence of both chronic and acute physical exercise on hepcidin [17,18,19]. In most studies of acute exercise, hepcidin levels increased: during military training [20], in rowers [21], or in female runners after a marathon [18]. This effect was associated with increases in inflammation and hemolysis induced by exercise [18]. The increase in hepcidin would probably have been followed by inhibition of both intestinal iron absorption and the release of iron from macrophages, both contributing to the development of iron deficiency in these athletes. Peeling et al. [22] examined the urinary hepcidin levels, inflammation process (the interleukin 6 and C-reactive protein levels), and iron metabolism during the 24 h after exercise. They concluded that high-intensity exercise was responsible for a significant increase in hepcidin levels, subsequent to a significant increase in IL-6 and serum iron. In the case of acute exercise, a relationship between this exercise modality and the acute phase response of inflammation has been demonstrated. This type of response is a complex of physiological processes occurring in response to some threats within the body, such as infection, trauma, the inflammatory process, strenuous exercise, or tissue injury [9].

The effects of chronic exercise on hepcidin levels have been studied less; there may be a tendency of the hormone concentrations to decrease. It seems that the duration of the training is not the most influential factor; rather, it is the volume of training (frequency, intensity) that has a major impact on iron levels. The maximum oxygen consumption (VO_2 max_) is defined as the highest rate of oxygen consumption that athletes may utilize during intense or maximal exercise. Dominguez et al. [23] proposed that an intensity of VO_2 max_ lower than 65% is not enough to trigger a response in iron metabolism and that higher values would start to increase hepcidin levels and impede iron absorption.

Our study is based on sport training for 120 days. We did not determine the hepcidin levels after a bout of exercise, but we took samples under resting conditions—in a context of long-term, regular exercise by our elite athletes, with a constant, mild intensity—and not in bouts of exercise that typically produce an increase in hepcidin levels. This is consistent with a study describing the effects of eight weeks of endurance running in female runners [24]. These authors analyzed the relationships among long-term exercise, hepcidin, and biomarkers of inflammation in a sample of 14 runners. In their study, serum hepcidin concentrations tended to decline during the running training and they did not find any change in C-reactive protein, used as a biomarker of inflammation. In this sense, we have previously reported the levels of different biomarkers of oxidative stress and inflammation in our triathletes [25]. The inflammation biomarkers tetranor-PGEM (tetranor prostaglandin E metabolite) and 11β-PGF_2α_ (11 β-prostaglandin F_2α_ metabolite) showed lower concentrations after the training program. If we consider the concept of the acute phase response, mentioned above, we may establish a connection between biomarkers of inflammation, acute exercise, and chronic exercise. The intensity of acute exercise can trigger an inflammatory response, as well as an increase in hepcidin levels. On the other hand, in long-term or chronic exercise, the body does not respond in the same way. It seems that, in our study, there was no inflammatory response to chronic exercise and this could explain the decrease in the levels of hepcidin. Kapasis et al. [26] also reported a reduction in inflammation with chronic training, due to the reduced release of pro-inflammatory cytokines, an increase in insulin sensitivity, an improvement of endothelial function, and a reduction in body weight.

As we mentioned before, the objective of this study was to determine the effects of the consumption of ACJ and chronic exercise on hepcidin levels—which can reflect iron status. We observed a slight and non-significant increase in hepcidin levels in the period of ACJ consumption, but these values did not differ statistically from those obtained with placebo ingestion. We know that the use of some supplements, as well as certain foods, may influence iron absorption, in contrast with the small and non-significant changes in hepcidin that we observed. Our ACJ is rich in polyphenol compounds, such as anthocyanins, as well as being a good source of ascorbic acid [27]. It has been demonstrated that polyphenols from the diet—found primarily in fruits and vegetables, spices, pulses, and cereals, and being especially abundant in tea, coffee, and red wine—reduce the absorption of iron in a dose-dependent manner. After the ingestion of foods containing phenolic compounds, these may form complexes with iron—impeding its absorption in the intestine [28,29]. Polyphenols are easily deprotonated at physiological pH levels in the presence of iron and form very stable complexes [30]. Metal ions with octahedral geometry, such as Fe^2+^ and Fe^3+^, can coordinate up to three catecholate or gallate groups, as reported in the excellent review of the basis of polyphenol compounds and iron binding by Perron and Brumaghim [31]. In a previous study conducted with beverages having different polyphenol concentrations, the more concentrated drinks, containing 100–400 mg of total polyphenols, reduced iron absorption by 60%–90%, whereas the drinks containing lower levels of polyphenols (20–50 mg) reduced iron absorption by 50%–70% [28]. In contrast, we did not observe changes in the levels of this hormone implicated in iron metabolism after the ingestion of our polyphenol-rich juice at the selected dosage (200 mL).

On the other hand, ascorbic acid is a potent promoter of iron absorption [32]. Dietary iron needs to be reduced from its ferric form (Fe^3+^) to the ferrous form (Fe^2+^) in order to be absorbed [33]. Thus, one of the routes to increase the absorption of iron is the consumption of reducing agents, such as ascorbic acid. It has been shown that ascorbic acid prevents the dose-dependent inhibitory effects of polyphenols (measured as tannic acid), as well as those of phytates [32]. These authors observed that ~50 mg of ascorbic acid would be necessary to improve the bioavailability of iron, to regular values, from a meal containing more than 100 mg of polyphenols (expressed as tannic acid); they suggested that 300 mL of orange juice would be adequate to restore the iron absorption from a meal containing 350 mg of polyphenols. Other authors have reported that red grape and prune juices high in polyphenols (>1 mg/mL) limited the bioavailability of iron to human colon cancer cells and the higher iron bioavailability was correlated with a high ascorbate/polyphenol ratio. These authors suggested a dietary balance of juices with a high polyphenol content, as well as a high ascorbate concentration (for proper iron absorption) [34].

The important contribution of the ascorbic acid provided by citrus-based juices is well known [35]. The ascorbic acid content of our juice was 30 mg/100 mL [36]. Iron absorption may have been enhanced slightly by high intake of ascorbic acid from our test ACJ; the mild increase in plasma hepcidin observed after ACJ intervention might be a consequence of a mild increment in the iron status of the organism. In this sense, further studies are needed to evaluate the real contribution of the different nutrients of juices, especially vitamin C, to hepcidin levels in order to provide advice regarding their intake in sport nutrition.

Finally, the serum ferritin concentration can be used to evaluate iron stores and it has been correlated with hepcidin in the general population [37], in elderly people [38], and in children [39]. We observed a tendency of the plasma ferritin levels to decrease in our triathletes during the training (data not shown), due to decreases in the iron levels. This effect has also been observed during the long-term training of runners [40]. Hence, the lower level of plasma iron due to exercise might attenuate an increase in hepcidin, as a homeostatic response.

## 4. Materials and Methods

### 4.1. Chemicals

The Dynabeads^®^ MyOne^TM^ Carboxylic Acid magnetic particles were obtained from Invitrogen (Life Technologies, Van Alley, CA, USA). The hepcidin standard was obtained from Peptide Institute Inc. (Osaka, Japan), *N*-ethyl-*N*′-(3-dimethylaminopropyl) carbodiimide hydrochloride (EDC) and 2-(*N*-morpholino)-ethane sulfonic acid (MES) from Calbiochem^®^ (Merck, Madrid, Spain), and phosphate-buffer saline (PBS) from Sigma Aldrich (Madrid, Spain). The solvents used, such as formic acid and acetonitrile, were LC-MS grade and were purchased from Panreac (Castelar del Vallés, Barcelona, Spain). The ultra-high quality (UHQ) water was produced using a Millipore water purification system (Madrid, Spain).

### 4.2. Instrumental—UHPLC-MS/MS Analysis

Chromatographic analyses of hepcidin levels in plasma were carried out with an ultra-high performance liquid chromatograph (UHPLC) coupled to a 6460 QqQ-MS/MS (triple quadrupole mass spectrometer) (Agilent Technologies, Waldbronn, Germany) equipped with an electrospray ionization (ESI) source. The method was based on that previously published by Bansal et al. [16], with some modifications. The separation of hepcidin was performed on an Acquity UPLC HSS T3 column (1.8 μm, 2.1 mm × 100 mm) (Waters, Milford, MA, USA). The mobile phases used were 0.1% formic acid in deionized H_2_O (A) and 0.1% formic acid in acetonitrile (B). The gradient started with 15% B, reached 90% B at 3 min, was maintained at 90% B for 5 min, and changed to 15% B at 5.01 min. The flow rate and injection volume were 0.2 mL·min^−1^ and 3 µL, respectively. The temperature of the column was maintained at 45 °C.

The mass spectrometry (MS) analysis was performed in the multiple reaction monitoring (MRM) ESI positive mode. The source optimized parameters were as follows: gas temperature: 300 °C, sheath gas temperature: 300 °C, sheath gas flow: 10 L·min^−1^, gas flow: 13 L·min^−1^, capillary voltage: 4500 V, and nebulizer pressure: 40 psi. The delta EMV was 400. The dwell time was 200 ms for all MRM transitions.

Data acquisition and processing were performed using MassHunter software version B.04.00 (Agilent Technologies).

Detection and quantification were performed using standard calibration curves of hepcidin at concentrations between 0.05 and 100 nM. For this purpose, we prepared different concentrations of standard hepcidin spiked in plasma and we followed the same protocol of extraction with the beads as for the test samples that were measured.

### 4.3. Extraction of Hepcidin from Human Plasma

Plasma samples (200 μL) were extracted for hepcidin purification and quantification. Plasma proteins were precipitated with two volumes of cold acetonitrile, the mixtures were centrifuged at 12,000× *g* for 10 min, and the whole supernatant was collected for analysis. Acetonitrile precipitates proteins and high-molecular-weight peptides whilst it does not affect hepcidin—which remains in the supernatant [41,42]. The concentration of total proteins was determined by the Bradford test, to optimize hepcidin extraction [43].

The subsequent extraction was performed according to the method of Bansal et al. [16], with some modifications. We employed Dynabeads^®^ MyOne^TM^ Carboxylic Acid nanoparticles (Invitrogen) to specifically capture molecules that are protein in nature. These beads are superparamagnetic, spherical particles, 1 μm in diameter and coated with carboxylic groups on the surface. Their small size, with a high surface area per mg, corresponds to a high ligand capacity. A carbodiimide is used to activate these beads, for amide bonding with primary amines of the peptide. After incubation, to allow affinity capture of hepcidin, the beads are applied to a magnet. The unwanted supernatant is removed and the beads washed to give the pure compound. The target hepcidin is eluted off the beads with conventional elution methods.

The effective isolation of the target molecule using magnetic beads is dependent on the bead concentration, target molecule size and concentration, the ligand′s affinity for the target molecule, concentration of total proteins, buffer used, and specific binding kinetics involved. Hence, we optimized the relative concentrations of each reagent and the quantity of plasma used and scaled the volumes needed for the best isolation of the target hepcidin from plasma samples.

After activation of the Dynabeads^®^ MyOne^TM^ Carboxylic Acid, following the manufacturer′s instructions, 100 μL were transferred to a 1.5-mL tube and left on the magnet for 2 min. After removing the supernatant, we added 120 μL of 15 mM MES buffer, pH 6.0, and vortexed the mixture. The supernatant was then removed by aspiration after a 2-min placement on the magnet. This MES buffer addition step was then repeated. The beads were re-suspended in 20 μL of 15 mM MES buffer, pH 6.0, activated with 20 μL of EDC (10 mg/mL), and incubated on a roller for 30 min at room temperature. Then, the tube was placed on the magnet for 2 min to remove the supernatant. The activated beads were added to the deproteinized sample plus 400 μL of 15 mM MES buffer, pH 6.0. The mixture was incubated on the roller for a minimum of 3 h.

The tube was left on the magnet for 2 min, to remove unbound proteins. A washing step was performed with 120 μL of PBS in 0.5% (*v*/*v*) Tween-20, by incubation on a roller for 10 min, followed by supernatant aspiration as described above. This step was repeated. Finally, the target compound—hepcidin—was eluted from the magnetic nanoparticles in 200 μL of acetonitrile/0.1% formic acid (1:4, *v*/*v*).

The efficacy of the extraction of hepcidin was tested at concentrations of 0.5 nM, 5 nM, and 10 nM, which are within the range of the values reported in the literature [16,22,42,44,45,46]. Hepcidin solution (20 μL) was added to the plasma before extraction with the beads, whilst in parallel another sample of plasma underwent extraction and then the same amount of hepcidin was added. The percentage recovery was calculated as (Area_PRE_/Area_POST_) × 100, where Area_PRE_ represents the samples with hepcidin added before bead treatment and Area_POST_ the samples with hepcidin added after bead treatment. The values obtained were above 90% in all cases.

### 4.4. Dietary Intervention

The functional aronia-citrus juice (ACJ) included in this study was a mixture of fresh citrus juice (95%) and 5% aronia extract (*Aronia melanocarpa*), based on a drink model developed before and reported by Gonzalez-Molina et al. [36]. The juice was produced on an industrial pilot scale by a company in the Murcia region [27]. The nutritional composition of the ACJ was: energy 38 kcal/100 mL; protein 0.45 g/100 mL; carbohydrates 9 g/100 mL; fat 0.03 g/100 mL. The vitamin C content of the juice was 30 mg per 100 mL. The contents of fruit flavonoids, hydroxycinnamates, and catechins are summarized in Table 2 [36].

The placebo beverage composition was based on a mixture of water, authorized red dye, flavoring agent, and sweetener. Its sensory characteristics were adjusted so that they were similar to those of the ACJ.

### 4.5. Sample Collection and Design of the Study

**Figure 3:**
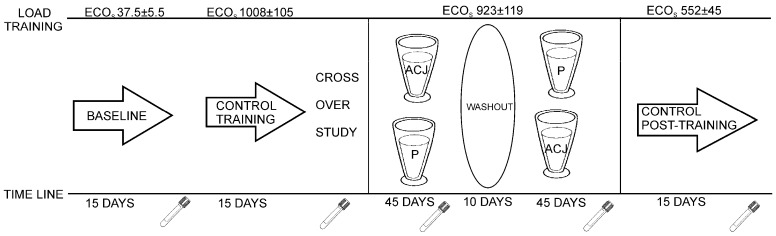
Study design.

The individuals involved in the sample collection were 16 Caucasian triathletes (six amateur training women and 10 elite training men), aged 19–21 years, from the University of Alicante (Alicante, Spain). They were non-smokers, had stable food habits, and did not receive any medication during the study. This study was conducted according to the guidelines laid down in the Declaration of Helsinki and all procedures involving human subjects were approved by the Bioethics Committee of the University Hospital of Murcia. Written, informed consent was obtained from all subjects. This study followed a randomized, double-blind, placebo-controlled crossover design (Figure 3).

The study design has been described previously and approved for the determination of the influence of ACJ on the generation of DNA oxidation catabolites [27]. Plasma samples as controls were collected before the diet supplementation with ACJ. The first control, baseline plasma, was collected after 15 days of training with minimal ECO loads (37.5 ± 5.5). The second control plasma was collected after a 15-day training of increased ECO loads (1008 ± 105) and this increase in ECO load was maintained until the end of the study. In the next stage, with the dietary intervention, the subjects were randomly divided into two groups and supplemented with either 200 mL of ACJ or 200 mL of placebo drink per day, for 45 days. Every day the intake of the drink took place 15 min after the athletes had finished their training, in order to improve the bioavailability of the phytochemicals from the ACJ [15]. After each intervention, 10 days were utilized as a washout period without drink intake, but the training was maintained. Subsequently, the intervention protocol was repeated, swapping the two groups according to the corresponding drink intake while maintaining their training (same ECOs). Afterwards, there was an ultimate phase of the study: 15 days without supplementation and with decreases of ECOs (control post-treatment).

Blood samples were collected at the end of each period in heparin sampling tubes, and centrifuged to separate the plasma from the cells.

The volunteers consumed a constant diet during the entire intervention, until its conclusion, to avoid any interference of the diet with the oxidative stress events. The diet was accurately designed and overviewed by nutritionists, using specific software for the calculation of the dietary parameters and caloric intake. The subjects were instructed to eat only the food provided to them by the nutritionists. Daily and weekly averages of the 24-h caloric intake were calculated (Table 3). The data were calculated using software available on the website http://www.easydiet.es, with the additional assistance of the Spanish (http://www.bedca.net/) and United States Department of Agriculture (USDA) (http://www.nal.usda.gov/fnic/foodcomp/search/) databases. All food for the study was prepared and weighed to achieve the desired and constant caloric level and intake of nutrients for each triathlete.

Anthropometric measurements, achieved according to the International Society of Advancement of Kinanthropometry (ISAK), were performed by the same, internationally-certified anthropometrist (level 2 ISAK), as described in Medina et al. [25].

The quantification of the training program was addressed to evaluate its effects on physiological adaptation and subsequent performance. In our work, the training load quantification was performed using the “objective-load scale” (ECOs) developed by Cejuela Anta and Esteve-Lanao [47]. The training load that a triathlete supports is an indication of his/her performance level. The method used allowed the quantification of the training loads in the sport of triathlon (swim, bike, run, and transitions), which are determined by the difficulty in maintaining technique, delayed muscle soreness, typical workout density, and energy cost of each separate sport. The values of daily and weekly trainings were determined and summarized to assess the training load (ECOs) of each volunteer, depending on their physical characteristics and the intensity of the training program (Table 4).

The “amateur athlete” term in this study refers to athletes that have a daily training load slightly lower than that of the elite athletes and a shorter time of training, but they have been monitored by a nutritionist and by a specialist in sport, so they are not the “amateur” that we commonly consider. Amateur training was performed by women, due to their intrinsic physiology (women on menstrual days could not follow an elite training schedule), and men followed an elite training schedule.

One limitation of the study is that we have combined the data of both genders and both types of training due to sample size reasons; amateur training is a very specific training load, with an exhaustive control of the volunteers. This strict control of nutrition, training, and rest helped us to homogenize the samples and decrease variability. The training was individualized for the volunteers according to their sex and physical condition. Hence, they have been pooled as a unique population following a very highly specialized training, in contrast with people that play sports occasionally, as a hobby, or less intensely.

### 4.6. Statistical Analysis

Hepcidin levels were determined in plasma samples of triathletes as nM. Box plots with quartiles (upper values, 75%; median, 50%; and and lower values, 25%) were generated. For the hepcidin concentrations, a Friedman′s non-parametric repeated measure analysis of variance (ANOVA) was used to compare the concentrations in the different groups, since the normality and/or equal variance tests failed. When a significant difference was found in the ANOVA, a pair-wise comparison was performed using the Wilcoxon signed-rank test with Bonferroni correction. For the statistical analyses, an adjusted value, *p* < 0.005, was considered to be significant. The statistical analyses were performed using the SPSS 17.0 software package (LEAD Technologies, Inc., Chicago, IL, USA).

## 5. Conclusions

Long-term, regular, and repetitive training caused significant decreases in the plasma hepcidin concentrations in elite triathletes. This effect may have arisen from a decrease in the inflammation status due to the long-term training, as well as being a homeostatic response to the decreased levels of iron—in order to enhance the absorption of this mineral. The ingestion of a juice made with aronia extract and citrus juice, rich in polyphenols, did not significantly affect the plasma hepcidin concentration, or at least not at the selected dose (200 mL). The high ascorbic acid content of this juice might attenuate the effect of polyphenols on iron absorption. Thus, this study—as a preliminary experiment—could help to design specialised sport food, like fruit juices or smoothies, to achieve a well-balanced diet in triathletes. Further studies are required to ascertain the time and conditions necessary to restore hepcidin levels, which reflect the iron status of triathletes.

## Figures and Tables

**Table 1:** Plasma concentrations of hepcidin in the different stages of the study. Data are shown in nM as mean ± standard deviation. Confidence intervals (CI) are included.

Groups	Mean	STD	Confidence Intervals (CI)
Baseline Control	116.6	63.3	78.29–154.83
Control 2-Exercise	78.7	71.1	44.23–124.91
Placebo Juice	15.2	12.6	5.60–38.04
Aronia-Citrus juice	29.4	19.6	18.61–40.27
Final Control	10.0	7.5	5.78–14.12

**Table 2:** Phenolic composition of aronia-citrus juice (per 100 mL).

	Mean	SD
**Flavanones (mg)**		
Eriocitrin	11.45	0.08
Hesperidin	13.54	0.14
**Flavones (mg)**		
Diosmetin-6,8-di-*O*-glucoside	7.75	0.19
Diosmin	<0.25	
Vicenin-2	0.59	0.02
**Anthocyanins (mg)**		
Cyanidin 3-*O*-glucoside	1.31	0.02
Cyanidin 3-*O*-arabinoside	9.19	0.20
Cyanidin 3-*O*-galactoside	15.08	0.10
Cyanidin 3-*O*-xyloside	1.11	0.03
Total Anthocyanins	26.70	0.35
**Hydroxycinnamic acids (mg)**		
Neochlorogenic acid	19.72	0.17
Chlorogenic acid	14.69	0.13
Σ Quercetin derivatives * (mg)	4.31	0.13

* Quercetin derivatives were quantified as the sum of quercetin 3-*O*-galactoside, quercetin-3-*O*-glucoside, and quercetin-3-*O*-rutinoside.

**Table 3:** Dietary parameters and caloric intake of the triathletes during the study.

	Mean	SD
Energy intake (kcal·d^−1^)	2446.3	528.5
Carbohydrate (g·d^−1^)	268.7	81.2
Dietary fibre (g·d^−1^)	21.4	8.3
Sugar (g·d^−1^)	100.9	28.8
Proteins (g·d^−1^)	108.5	35.6
Total lipids (g·d^−1^)	110.4	4.7
SFA ^a^ (g·d^−1^)	31.6	2.8
MUFA ^b^ (g·d^−1^)	56.7	0.1
PUFA ^c^ (g·d^−1^)	16.4	0.7
Vitamin C (mg·d^−1^)	156.9	30.9
Vitamin A (µg·d^−1^)	2198.7	1090.8
Vitamin E (mg·d^−1^)	17.5	5.0
Vitamin D (mg·d^−1^)	869.9	167.2
Iron (mg·d^−1^)	17.9	4.2
Selenium (mg·d^−1^)	126.4	33.1

^a^ Saturated fatty acids; ^b^ Monounsaturated fatty acids; ^c^ Polyunsaturated fatty acids.

**Table 4:** Baseline physical characteristics of the triathletes (*n* = 16).

	Mean	SD
Weight (kg)	62.3	10.5
Height (m)	1.7	0.1
BMI ^a^ (kg·m^−2^)	21.7	0.7
Total fat (kg)	9.0	0.4
Lean weight (kg)	26.1	7.5
Subescapular skinfold (mm)	11.2	2.2
Tricipital skinfold (mm)	12.6	5.2
Bicipital skinfold (mm)	7.9	3.5
Ileocrestal skinfold (mm)	15.9	5.4
Supraespinal skinfold (mm)	11.7	3.7
Abdominal skinfold (mm)	19.8	4.7
Thigh skinfold (mm)	21.1	8.7
Calf skinfold (mm)	11.9	4.1

^a^ Body mass index.
